# Using Small-Area Estimation to Describe County-Level Disparities in Mammography

**Published:** 2009-09-15

**Authors:** Karen L Schneider, Kate L. Lapane, Melissa A Clark, William Rakowski

**Affiliations:** John Snow, Inc. Dr Schneider was affiliated with the Department of Community Health, Brown University, Providence, Rhode Island, at the time of the study; Virginia Commonwealth University, Richmond, Virginia; Brown University, Providence, Rhode Island; Brown University, Providence, Rhode Island

## Abstract

**Introduction:**

Breast cancer control efforts could benefit from estimating mammography prevalence at the substate level because studies have primarily analyzed health survey data at the national and state levels. The purpose of this study was to evaluate the extent to which geographic disparities exist in mammography use across counties in the contiguous United States.

**Methods:**

We estimated county-level prevalence of recent mammography (past 2 years) for women aged 40 to 79 years by using synthetic regression, a small-area estimation method. The 2000 Behavioral Risk Factor Surveillance System (BRFSS), 2000 Census, Area Resource File, and Food and Drug Administration mammography facility data were merged by BRFSS respondents' county of residence. We conducted separate analyses to produce county-level prevalence estimates for each race and age group.

**Results:**

Mammography use varied geographically, and the magnitude of geographic disparities differed by race and age. Nonwhite women showed the lowest prevalence of mammography and widest range in county-level estimates. Women aged 40 to 49 had generally lower prevalence than other age groups, while women aged 65 to 79 showed the greatest variation in county-level mammography estimates.

**Conclusions:**

Small-area estimation using BRFSS data is advantageous for surveillance of mammography use at the county level. This method allows documentation of geographic disparities and improves our understanding of the spatial distribution of mammography prevalence. Future interventions should consider this county-level geographic variation, targeting women in the neediest counties.

## Introduction

The Centers for Disease Control and Prevention (CDC) designed the Behavioral Risk Factor Surveillance System (BRFSS) to produce state-level estimates of risk factors and health behaviors. The BRFSS collects information about respondents' county of residence, making county-level analyses possible. Recently, public health practitioners have been interested in obtaining BRFSS data on the substate level; however, the small county-level samples often produce unstable prevalence estimates with large standard errors. CDC recommends a minimum of 50 observations to reliably estimate prevalence (1).

Breast cancer control is a research area that could benefit from estimating prevalence at the substate level. Most of the data available on the use of preventive services, like mammography, are national and state data, and studies have identified correlates of cancer screening on these geographic levels by using national health survey data (2,3). Research is needed on subpopulations defined by geographic units smaller than the state because findings from the national or even state level often do not translate to the contextual experience of women on the county or neighborhood level (4). Substate variation in mammography prevalence (5,6) has been found for metropolitan statistical areas and counties with adequate sample size (≥50). Geographic gaps exist in mammography interventions; certain areas disproportionately receive funds and outreach (7). Women who are not screened may be concentrated in locations with particular screening barriers (8).

Data aggregation strategies attempt to overcome sample-size limitations by combining several years of data for 1 county (9) or combining data from several neighboring counties (3). However, temporal aggregations conceal time-trend differences, and spatial aggregations limit the ability to show interarea differences. An alternative method is small-area analysis, a statistical procedure that aims to produce stable estimates for areas of inadequate sample size. Studies have used various small-area estimation techniques to estimate prevalence at the substate level (10,11). However, a recent study found that synthetic multilevel regression produced the most valid prevalence estimates (1).

The purpose of this study was to apply the synthetic regression estimation technique to determine the extent to which geographic disparities exist in mammography and the extent to which the magnitude and distribution of geographic disparities vary by race and age. One of the national health objectives related to public health infrastructure is to ensure that health agencies have the necessary infrastructure by increasing the proportion of leading health indicators for which data are available (12). If data are not available to state and local public health agencies, then health or access problems may not be identified for a population or distinct subpopulations of an area (13). Local health departments can use small-area analysis for their data needs at these local levels and for select populations in these areas to identify disparities and target interventions and public health funding.

## Methods

### Data sources

The BRFSS is an annual random-digit–dialed survey of noninstitutionalized adults (≥18 years) that is funded by CDC and executed by health departments in every state and the District of Columbia (14). We used BRFSS data from 2000, even though more recent BRFSS data are available, to allow temporal comparability with other data sources.

Information on county-level sociodemographic and health care measures came from the 2000 US Decennial Census (15) and the Area Resource File (16). Mammography facility data certified by the Food and Drug Administration (FDA) (17) provided a county-level measure of access to mammography facilities. We merged and linked data sets by BRFSS respondents' county of residence.

### Study sample

The analyses included women aged 40 to 79 years. An age threshold of 40 years captured the most inclusive screening guidelines provided by the American Cancer Society and the US Preventive Services Task Force (18,19). We selected an age limit of 79 years because the benefit of screening for older women is debatable (20,21). For these analyses, the age range of the BRFSS sample matched the age categories of the census data.

Of the 58,901 women aged 40 to 79 who participated in the 2000 BRFSS, we excluded from the analyses women with missing information on county code (n = 14,599; 24.8% of initial sample), who refused to provide their county of residence (n = 167; 0.3% of initial sample), and with "don't know" or "not sure" responses to county of residence (n = 140; 0.2% of initial sample). A large percentage of women were missing information on county of residence because CDC set the county code as missing for women from counties with fewer than 50 observations in the 2000 public-use BRFSS data. Thus, the final analytic sample included 43,995 women with valid data on mammography history who lived in the continental United States and had Federal Information Processing Standard (FIPS) county codes to link with census data.

### Dependent variable

The women's health section of the BRFSS core questionnaire included questions about breast cancer screening. We coded recent mammography status as a dichotomous variable. Women who reported having had a mammogram in the past 2 years were coded as recently screened. Women who reported never having had a mammogram or that their most recent mammogram was more than 2 years ago were coded as not recently screened. We coded women who answered "don't know" or refused to answer the mammography questions (n = 349) as not recently screened, consistent with other studies (5).

### Independent variables

A cross-classification of race (white, black, or other nonwhite race) and age (40-49, 50-64, or 65-79 y) was the primary independent variable from the BRFSS data. We selected race and age because counties vary in sociodemographic composition across the United States (22), recent mammography is related to these 2 variables in bivariate analyses, and the same cross-classification of variables is possible with census data.

We treated Hispanic ethnicity as a confounder because bivariate analyses showed that the prevalence of mammography was lower among Hispanics. Additionally, Hispanic ethnicity was not included in our cross-classification of race and age because it was not possible to use census data to create a cross-classification of race, ethnicity, *and* age for noninstitutionalized women aged 18 years or older.

We also considered county-level variables shown to be related to recent mammography as confounders, similar to variables explored by Legler et al (23). We used scatterplots and correlation coefficients to evaluate collinear relationships among independent variables. The final model included average per capita annual household income (census data), number of doctors per 10,000 women aged 40 or older and Rural-Urban Continuum Codes (Area Resource File data), and number of FDA-certified mammography facilities per 10,000 women aged 40 or older (FDA data). We coded all county-level variables as categorical variables on the basis of quintiles of their distributions because they were linearly associated with the outcome.

### Statistical analyses

Small-area estimation by synthetic regression was a 2-step process. First, we used generalized estimating equations for logistic regression to analyze the association between recent mammography and the race-age variable, producing a predicted prevalence for each race-age category. The model accounted for the clustering of women by county and the complex BRFSS survey design and included both individual- and county-level confounders:

Logit(YIJK)=β0+β1(Raceijk*Ageijk)+β2(Hispanicijk)+β3Vjk

where Y_ijk_ is recent mammography for women (i) who reside in counties (j) in states (k); Race_ijk_*Age_ijk_ is the cross-classification of the race and age categorical variables for women (i) nested within counties (j) and states (k); Hispanic_ijk_ is Hispanic ethnicity for women (i) nested within counties (j) and states (k); V_jk_ is a vector of county (j) level variables nested within the state (k); β_0_ is the population-averaged, or marginal, intercept term; β_1_ is the population-averaged coefficient for the race*age cross-classification; β_2_ is the population-averaged coefficient for Hispanic ethnicity; and β_3_ is the vector of population-averaged coefficients for county-level variables. We built separate models for each regional census division to account for regional effects when predicting mammography prevalence.

Next, we used the resulting predicted probabilities from step 1 in the synthetic estimation of mammography prevalence to estimate the proportion of women in each county who reported recently having had a mammogram. The synthetic estimate of the prevalence (p_*j*_) of recent mammography for county *j* equaled

pj=Σ(nij/nj)pij

where p*_ij_* is the predicted probability for a specified race-age category from step 1; n_*ij*_ is the number of women in race-age group (i) who reside in the county (j); and n_*j*_ is the total number of women aged 40 to 79 who reside in the county (j). The sum of the race-age weighted prevalences equaled the prevalence of recent mammography for the county.

We performed all statistical analyses by using SAS version 9 (SAS Institute, Inc, Cary, North Carolina) and SAS-Callable SUDAAN version 9.1 (Research Triangle Institute, Research Triangle Park, North Carolina) to adjust for complex survey design. We used small-area analysis to estimate the prevalence of recent mammography in each county for women aged 40 to 79. Further, we repeated the procedure to estimate county-level mammography prevalence for each race and age category to determine the extent to which geographic disparities existed for and differed by sociodemographic subgroups.

We mapped results using ArcGIS version 9.2 (ESRI, Redlands, California), which produced a visualization of county-level prevalence to identify geographic disparities in screening. For the maps, the natural-breaks classification method determined the prevalence categories of recent mammography (24).

We hypothesized that county-level analyses would produce similar patterns in prevalence as found at the state level, that counties in the Northeast would report the highest prevalence of mammography, and that counties in the Southeast and Mountain regions would report the lowest prevalence. A prior study found similar geographic patterns when using 2002 BRFSS data to estimate prevalence of mammography for the 9 census regions (5). We posited that some intrastate variation in mammography prevalence would be observed and that the magnitude and location of these geographic disparities would vary by race or age. We further hypothesized that counties with a greater proportion of younger and nonwhite women would have lower prevalence of mammography because these groups have historically had lower prevalence of mammography (25) and are not uniformly represented across US counties (22).

### Evaluation of small-area estimates

Because a gold standard was unavailable, we used state-level direct estimates to evaluate the validity of small-area estimates. We aggregated county-level prevalence estimates of recent mammography that were obtained from the small-area analysis to the state level. We also directly estimated state-level prevalence of recent mammography by using 2000 BRFSS data. State-level aggregated small-area estimates were compared with state-level direct prevalence estimates, and we calculated the average differences between them, as well as the 95% confidence intervals (CIs). We calculated Pearson correlation coefficients between the 2 measures and then confirmed the correlations by conducting bivariate linear regression analyses. *P* values < .05 were considered significant.

## Results

The prevalence of recent mammography in our national sample of women aged 40 to 79 years was 78.5%. The average county-level prevalence of recent mammography was 77.9% (95% CI, 77.8%-78.0%) (Table 1); small-area estimates on the county level ranged from 69.6% to 84.0%. We found county prevalence estimates calculated by using small-area analysis to be generally reliable. Overall, 99.6% of counties had 95% confidence intervals within 5 percentage points of the estimates. For example, the prevalence of recent mammography for women aged 40 to 79 in Stephens County, Georgia, was 80.0% (95% CI, 78.9%-81.0%). The largest confidence interval for a county-level prevalence estimate (59.3%-79.9%) was in Shannon County, South Dakota.

The highest prevalence of mammography in our study sample was in the New England, North and South Atlantic, and East North Central census divisions (Figure). The lowest prevalence of mammography was in the Mountain states and Texas. Intrastate variation was greatest in California, where certain counties were in the lowest category of prevalence (69.6%-74.3%) and others were in the highest category (≥81.6).

**Figure.**
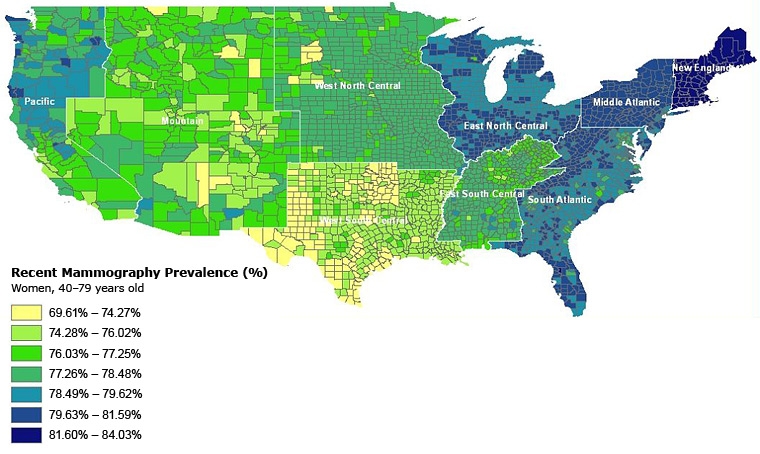
County-level prevalence of recent mammography for women aged 40 to 79 years in the continental United States. Estimated by using data from the 2000 Behavioral Risk Factor Surveillance System and US Decennial Census.

County-level prevalence estimates varied by race (race- and age-specific maps not shown). For white race, the average county-level prevalence was 78.4%, and county-level estimates ranged from 73.8% to 84.3%. Lower-prevalence areas clustered in Texas and the Mountain states, and areas on the high end of the distribution clustered in the Pacific, New England, and Atlantic divisions.

The average county-level prevalence was 78.5% for black women and 67.4% for women of other nonwhite race. While the average county-level prevalence for black women was nearly equal to the prevalence for white women, the prevalence for other nonwhite women was 10 percentage points lower. The range in screening prevalence across US counties was higher for nonwhite women than for white women (black range, 65.0%-92.0%; other nonwhite range, 36.0%-83.0%). For black women, counties in the Mountain division, upper peninsula of Michigan, and Wisconsin had the lowest prevalence of mammography (65.0%-72.6%), and counties in the Pacific, New England, and West North Central divisions had the highest prevalence (86.8%-92.0%). Prevalence of recent mammography was generally lower for other nonwhite women but was highest in counties of the East North Central and New England divisions (75.4%-83.0%) and lowest in counties of the South Central divisions (36.0%-62.9%).

The average county-level prevalence was highest for women aged 50 to 64 (82.3%), followed by women aged 65 to 79 (80.7%), and then lowest for women aged 40 to 49 (71.2%). Although women aged 40 to 49 had on average the lowest county-level prevalence of recent mammography, the largest range in county-level prevalence estimates was seen in women aged 65 to 79  (range, 53.0%-88.0%). Texas, the south Mountain division, and southern California had the lowest screening prevalence for women aged 40 to 49 (56.7%-67.4%), while the New England and East North Central divisions had the highest prevalence of mammography for women in this age group (75.8%-79.8%). Intrastate variation in prevalence existed in North and South Dakota. Prevalence of recent mammography for women aged 50 to 64 was high (75.7%-89.5%); areas of highest prevalence were found in the New England and South Atlantic states (85.1%-89.5%). The Southern California, Mountain, and parts of the South Central divisions had the areas of lowest prevalence for this age group (75.7%-80.2%). Finally, the Pacific, Mountain, New England, and parts of the South Atlantic divisions had the highest prevalence of recent mammography for women aged 65 to 79 (84.0%-88.0%). Counties in the West North Central census division and parts of Texas had the lowest prevalence of mammography for women in this oldest age group (53.0%-77.9%).

On average, small-area estimates aggregated to the state level were within 2.4 percentage points of the directly derived state estimates of recent mammography prevalence from the BRFSS (range, −10.3 to 8.6) (Table 2). Aggregated small-area estimates were generally higher, but correlated, with direct estimates (ρ = 0.58; *P* < .001; regression β = 0.34; *P* < .001). The largest difference between direct state estimates and aggregated small-area estimates occurred in Idaho, where the aggregated value overestimated the direct value by 10.3 percentage points. The smallest differences occurred in Kansas, New York, and North Carolina, where the aggregated value underestimated the direct value by 0.3 percentage points.

## Discussion

We demonstrated the value of small-area analysis in producing county-level prevalence estimates of mammography for women aged 40 to 79 years. The prevalence of recent mammography across counties in the continental United States is generally high. Our findings were consistent with estimates calculated for metropolitan statistical areas by using BRFSS data from a similar time period (9). Furthermore, we showed that small-area estimates aggregated to the state level were comparable to state-level direct estimates of recent mammography prevalence.

Mapping of prevalence estimates showed that the location and magnitude of screening disparities varied by race and age. County-level prevalence estimates and lower minimum prevalence values varied more for black and other nonwhite women than for white women. Research shows substantial disparities in the quality and quantity of health care received by minority populations (26), and these disparities are disproportionately distributed across the nation (27). Regional variations in mammography use by race are likely due to quality of care, insurance coverage, and availability of health care to minority populations (28). By monitoring mammography use in minority populations and tracking these geographic variations, interventions aimed at improving quality and access can be targeted to the neediest counties (23).

Our age-related findings may be likely explained by confusion over conflicting screening recommendations for younger and older women. In 2000, the benefit of mammography for women younger than 50 was being debated because the reduction in breast cancer mortality for these women was uncertain, given their low incidence of cancer (19,29). Similar questions arose concerning the benefits of screening for older women, who commonly have comorbidities and lower life expectancy (20,21). The range of prevalence estimates for women aged 40 to 49 (56.7%-79.8%) was generally lower than for other age groups, and range in county-level prevalence estimates was largest among women aged 65 to 79 (53.0%-88.0%). Women in these age categories may differentially be recommended for mammography by clinicians or have misperceptions about their need for screening. A recent study found that among women with access to health care, nonscreeners were more likely to be younger (aged 40-49), and these younger women were more likely to have postponed having a mammogram than to not realize that they needed the examination (30). Additionally, among women aged 65 or older with access to health care, 75% reported no mammography recommendation from a physician in the past year (30). Knowledge of age-related geographic disparities could help public health professionals in underscreened counties develop programs targeted to 2 groups: women who might not perceive the benefit of mammography at their age and clinicians who might not be recommending or referring younger and older women to screening.

Findings showed intrastate variation in county-level prevalence of mammography. Conducting substate surveillance for subpopulations (eg, by race or age) to identify interregional and intrastate differences is vital to state and local health officials' understanding of who remains underscreened for breast cancer (9). This knowledge can be used to design interventions and formulate policies to increase screening in high-risk areas, subsequently reducing rates of breast cancer mortality and late-stage diagnosis (31).

In general, we observed little variability in the small-area estimates for women aged 40 to 79, probably because of the selection of a cross-classification variable. Unfortunately, a race-age-sex cross-classification was the only combination of variables possible using publicly available census data for the noninstitutionalized adult population. Previous studies identified race and age as determinants of screening use (32,33), but income and insurance/usual provider are stronger determinants of screening (34). If our cross-classification variable could include income and insurance/usual provider, then we would probably have seen more variation in small-area screening prevalences, especially since the relationship between income, insurance/usual provider, and screening varies by region (9). Even with this attenuation, small-area estimation is necessary to understand geographic disparities in mammography use if the results are reported appropriately. The results are valuable for surveillance purposes and tracking trends over time because these are often the only data available on the substate level (35).

Small-area estimation was necessary because 92% of US counties (using 2000 BRFSS data) had insufficient sample size to estimate the prevalence of mammography. CDC in recent years has increased the sample size of the BRFSS to support substate or subpopulation analyses (6). From 2000 to 2006, the number of women aged 40 to 79 who were interviewed increased from 62,389 to 146,156. Even with the BRFSS sample size increase, small-area estimation methods would be needed to estimate mammography prevalence in 80% of US counties in 2006. CDC should continue increasing the BRFSS sample size by recommending target sample-size goals and providing funding to states.

This study has several potential biases and limitations. The political and conceptual definitions of county differ by state. In the BRFSS, the parishes, boroughs, or counties defined within a state are identified by using FIPS county codes. However, public health departments, outreach, and interventions do not occur on these levels in all states. For example, some states, such as Rhode Island, have regional or state health departments rather than county health departments, and thus county data mean little or nothing in terms of public health activities in these states. These different meanings of "county" may have introduced random error into our results. Additionally, questions on the BRFSS do not allow differentiation of screening mammograms from diagnostic mammograms; thus, BRFSS data may overestimate the prevalence of screening mammography.

This study excluded 14,906 women with no information on FIPS county code to link with census data because CDC did not include county code in the BRFSS for people residing in counties with fewer than 50 observations. The purpose is to prevent the public from analyzing and reporting prevalence estimates for counties with inadequate sample size. In our exploratory analyses, compared with included women, women excluded from the analyses were more likely to be non-Hispanic white, married, and educated (college graduates). While it is uncertain how our small-area estimates would differ with the inclusion of these women, the estimates would probably be slightly higher because education, marriage, and white race are all positively associated with mammography use (2).

Finally, data collected for the BRFSS are self-reported. Research generally shows self-reported cancer screening history to be valid (36). However, mammography was found to be overreported in the BRFSS by racial/ethnic minority women when Medicare claims data were used as the standard (37), and thus BRFSS-derived prevalence of mammography for minority women may be overestimated. Furthermore, prior studies found that women tend to underestimate time since their last mammogram ("telescoping"), which could also contribute to overestimation of BRFSS data (38).

In conclusion, small-area estimation using BRFSS data is advantageous for surveillance of mammography at the county level. This method allowed us to document geographic disparities and improve knowledge of the spatial distribution of mammography prevalence. Future interventions should consider this county-level geographic variation to target women in the neediest counties.

## Figures and Tables

**Table 1:** County-Level Prevalence Estimates of Mammography Produced From Small-Area Estimation by Using BRFSS and Census Data, Women Aged 40-79 Years, by Race and by Age, 2000

**Category**	Mean^a^ County-level Prevalence, % (95% CI)	Range of County-level Prevalence, %
**Overall**	77.9 (77.8-78.0)	69.6-84.0
**Race**		
White	78.4 (78.4-78.5)	73.8-84.3
Black^b^	78.5 (78.4-78.6)	65.0-92.0
Other nonwhite^c^	67.4 (67.2-67.7)	36.0-83.0
**Age, y**		
40-49	71.2 (71.0-71.3)	56.7-79.8
50-64	82.3 (82.2-82.3)	75.7-89.5
65-79	80.7 (80.6-80.8)	53.0-88.0

Abbreviations: BRFSS, Behavioral Risk Factor Surveillance System; CI, confidence interval.

a Mean and median of county-level prevalence were <2 percentage points different.

b Three hundred forty-two counties had no black population for which to estimate prevalence.

c Four counties had no other nonwhite race population for which to estimate prevalence.

**Table 2:** Comparison of Directly Estimated State Prevalence of Recent Mammography for Women Aged 40-79 Years With County-Level Small-Area Estimates Aggregated to the State Level, BRFSS, 2000

State	Direct Estimates, %	Aggregated Small-area Estimates, %	Percentage Point Difference
Alabama	76.3	77.6	−1.3
Arizona	80.9	76.6	4.4
Arkansas	73.8	75.6	−1.8
California	78.6	77.3	1.3
Colorado	75.4	76.2	−0.8
Connecticut	84.8	83.5	1.3
Delaware	88.0	79.4	8.6
District of Columbia	82.0	78.2	3.9
Florida	78.9	79.9	−1.0
Georgia	77.4	79.3	−1.9
Idaho	66.4	76.7	−10.3
Illinois	77.0	79.6	−2.6
Indiana	74.3	79.6	−5.3
Iowa	79.6	77.7	1.9
Kansas	77.8	77.5	0.3
Kentucky	77.8	77.1	0.6
Louisiana	76.9	75.2	1.8
Maine	79.9	83.8	−3.9
Maryland	82.0	79.1	2.9
Massachusetts	84.9	83.4	1.5
Michigan	83.7	79.6	4.2
Minnesota	75.4	77.5	−2.1
Mississippi	73.5	77.8	−4.3
Missouri	77.2	77.8	−0.6
Montana	76.2	76.7	−0.5
Nebraska	79.7	77.6	2.1
Nevada	74.1	76.5	−2.5
New Hampshire	78.7	83.7	−4.9
New Jersey	77.4	79.4	−2.1
New Mexico	77.0	76.2	0.8
New York	80.3	80.0	0.3
North Carolina	79.7	79.4	0.3
North Dakota	78.6	77.3	1.2
Ohio	82.1	79.6	2.5
Oklahoma	72.8	74.2	−1.4
Oregon	77.5	78.7	−1.2
Pennsylvania	78.9	80.2	−1.3
Rhode Island	84.6	83.5	1.1
South Carolina	79.8	79.3	0.5
South Dakota	77.2	76.8	0.4
Tennessee	78.5	77.3	1.2
Texas	72.6	74.5	−1.9
Utah	74.5	76.5	−2.0
Vermont	79.0	83.7	−4.7
Virginia	81.2	79.4	1.8
Washington	77.3	78.3	−1.0
West Virginia	72.7	79.9	−7.1
Wisconsin	79.2	79.5	−0.4
Wyoming	67.8	76.4	−8.6

Abbreviation: BRFSS, Behavioral Risk Factor Surveillance System.
