# Erratum, Vol. 6: Issue 4

**Published:** 2009-12-15

In the article "Using Small-Area Estimation to Describe County-Level Disparities in Mammography," Table 2 contained an unnecessary footnote. The correction was made to our Web site on November 5, 2009, and appears online at http://www.cdc.gov/pcd/issues/2009/oct/08_0210.htm. We regret any confusion or inconvenience this error may have caused.

